# The effects of external Mn^2+^ concentration on hyphal morphology and citric acid production are mediated primarily by the NRAMP-family transporter DmtA in *Aspergillus niger*

**DOI:** 10.1186/s12934-020-1286-7

**Published:** 2020-01-30

**Authors:** Balázs Fejes, Jean-Paul Ouedraogo, Erzsébet Fekete, Erzsébet Sándor, Michel Flipphi, Áron Soós, Ákos P. Molnár, Béla Kovács, Christian P. Kubicek, Adrian Tsang, Levente Karaffa

**Affiliations:** 1 Department of Biochemical Engineering, Faculty of Science and Technology, University of Debrecen, Egyetem tér 1, Debrecen, 4032 Hungary; 2 Juhász-Nagy Pál Doctoral School of Biology and Environmental Sciences, University of Debrecen, Debrecen, Hungary; 3 Centre for Structural and Functional Genomics, Concordia University, Montreal, QC Canada; 4 Institute of Food Science, Faculty of Agricultural and Food Science and Environmental Management, University of Debrecen, Böszörményi út 138, Debrecen, 4032 Hungary; 5 Institute of Chemical, Environmental and Bioscience Engineering, TU Wien, Vienna, Austria

**Keywords:** *Aspergillus niger*, Manganese ions, Citric acid, Manganese transporter

## Abstract

**Background:**

Citric acid, a commodity product of industrial biotechnology, is produced by fermentation of the filamentous fungus *Aspergillus niger*. A requirement for high-yield citric acid production is keeping the concentration of Mn^2+^ ions in the medium at or below 5 µg L^−1^. Understanding manganese metabolism in *A. niger* is therefore of critical importance to citric acid production. To this end, we investigated transport of Mn^2+^ ions in *A. niger* NRRL2270.

**Results:**

we identified an *A. niger* gene (*dmtA*; NRRL3_07789), predicted to encode a transmembrane protein, with high sequence identity to the yeast manganese transporters Smf1p and Smf2p. Deletion of *dmtA* in *A. niger* eliminated the intake of Mn^2+^ at low (5 µg L^−1^) external Mn^2+^ concentration, and reduced the intake of Mn^2+^ at high (> 100 µg L^−1^) external Mn^2+^ concentration. Compared to the parent strain, overexpression of *dmtA* increased Mn^2+^ intake at both low and high external Mn^2+^ concentrations. Cultivation of the parent strain under Mn^2+^ ions limitation conditions (5 µg L^−1^) reduced germination and led to the formation of stubby, swollen hyphae that formed compact pellets. Deletion of *dmtA* caused defects in germination and hyphal morphology even in the presence of 100 µg L^−1^ Mn^2+^, while overexpression of *dmtA* led to enhanced germination and normal hyphal morphology at limiting Mn^2+^ concentration. Growth of both the parent and the deletion strains under citric acid producing conditions resulted in molar yields (Y_p/s_) of citric acid of > 0.8, although the deletion strain produced ~ 30% less biomass. This yield was reduced only by 20% in the presence of 100 µg L^−1^ Mn^2+^, whereas production by the parent strain was reduced by 60%. The Y_p/s_ of the overexpressing strain was 17% of that of the parent strain, irrespective of the concentrations of external Mn^2+^.

**Conclusions:**

Our results demonstrate that *dmtA* is physiologically important in the transport of Mn^2+^ ions in *A. niger*, and manipulation of its expression modulates citric acid overflow.

## Background

Manganese is a trace element that is essential for all organisms [1]. It serves as a cofactor for the reactions catalysed by metalloproteins including DNA and RNA polymerases, peptidases, carboxylases, superoxide dismutase, sugar transferases and the water oxidation complex in photosystem II (reviewed by Reddi et al. [2]). The availability of manganese for the cell is therefore essential [3].

In fungi, manganese deficiency has been shown to result in alterations in hyphal morphology and reduction of sporulation [4]. In *Aspergillus niger*, manganese deficiency results in elevated production and excretion of citric acid [4, 5], which today is the exclusive industrial process for the production of this metabolite. To reach high yields, the manganese concentration in the medium must be kept at or below 5 μg L^−1^, which exceeds the amount bound as contaminant to the carbon source required for this fermentation [6]. Consequently, manganese ions need to be removed from the fermentation broth (by cation exchanging of the carbon source solution or by precipitation with hexocyanoferrate), prevented from intake by addition of copper, or counteracted by addition of alcohols and other compounds [7, 8]. Yet another, still hypothetical, way to eliminate the detrimental effect of manganese on citrate production is the modulation of manganese transport activity.

The import of manganese into cells is mediated by transporters. The divalent metal transporter 1 (DMT1), a member of the NRAMP (Natural Resistance-Associated Macrophage Proteins) transporter family (protein family PF01566; transporter classification TC 2.A.55), is the primary Mn^2+^ transporter in mammalian cells, although several other transmembrane proteins have also been described to import Mn^2+^ in mammals [3]. The driving force for the metal ion transport is proton gradient (proton-motive force). In *Saccharomyces cerevisiae*, two NRAMP transporters (named Smf1p and Smf2p) have been shown to be responsible for modulating intracellular Mn^2+^ levels: Smf1p, responsible for maintaining the intracellular manganese levels required for its anti-oxidant action; and Smf2p which imports manganese for the Mn-requiring enzymes mentioned above [2, 9]. Orthologues of the *SMF1/2* genes have been identified and studied in few fungi, including the fission yeast *Schizosaccharomyces pombe* [10, 11], the basidiomycete yeast *Cryptococcus neoformans* [12] and the white-rot basidiomycete *Phanerochaete sordida* [13]. To date, the only filamentous fungus of the subphylum Pezizomycotina in which a NRAMP transporter has been studied is the endophyte *Exophiala pisciphila*; but Mn^2+^ transport or -homeostasis was not assessed [14]. Hockertz et al. [15] described the presence of a high-affinity Mn^2+^-permease in *A. niger* which also transports Zn^2+^, Cu^2+^ and Cd^2+^, but the encoding gene has not been identified and it is therefore not known whether it is a member of the NRAMP transporter family.

In this paper, we have identified and characterized a single NRAMP-family permease of *A. niger* (DmtA) that has high sequence identity to both Smf1p and Smf2p. We show here that manipulation of *dmtA* gene activity, by gene deletion and gene overexpression, has a significant impact on the interplay between the extracellular manganese concentration, citrate production and morphological development in this fungus.

## Results

### In silico identification of the putative divalent metal ion transporter *dmtA* in Aspergillus niger

A BLASTP search of the *A. niger* genome with the *S. cerevisiae* Smf1p and Smf2p sequences as queries resulted in the identification of NRRL3_07789. The encoded protein comprises 575 amino acids and exhibits 58% amino acid identity with both yeast orthologues. Typical for fungal NRAMP divalent metal/proton symporters, NRRL3_07789 forms 11 predicted transmembrane helices. This gene is present in the parent of NRRL2270, *A. niger* ATCC 1015 (JGI protein ID Aspni7:1110874), and the glucoamylase producer *A. niger* CBS 513.88 (JGI protein ID Aspni_DSM_1:159254). The corresponding proteins share 100% amino acid sequence identity. Their chromosomal environment is also completely syntenic within ± 100 kb (data not shown). From these observations we conclude that neither the *dmtA* gene nor its genomic locus has been altered in proficient citric acid producing strains.

### NRRL3_07789 encodes a transporter capable of high-affinity Mn^2+^ ion transport

To demonstrate that NRRL3_07789 encodes an *A. niger* divalent metal ion transporter capable of manganese transport, we first set up a system for measuring the rate of transport of Mn^2+^ into the cells by monitoring the decrease of Mn^2+^ concentration in the medium. Control experiments with the parental strain showed that the intake rate was linear within the first 24 h of cultivation (samples were taken every 3 h) and within biomass concentrations of between 0.1 and 0.5 g L^−1^, and that only negligible amount of Mn^2+^ was bound to the cell walls (Additional file 1: Fig. S1 and Additional file 2: Table S1). Under these conditions, *A. niger* exhibited a maximal intake rate of 10 ± 2 pmol min^−1^ g_DCW_^−1^ at 100 μg L^−1^ of Mn^2+^. This corresponds well to the 6.12 ± 0.49 pmol min^−1^ g_DCW_^−1^ determined by Hockertz et al. [15] using a radiolabelled method.

**Fig. 1:**
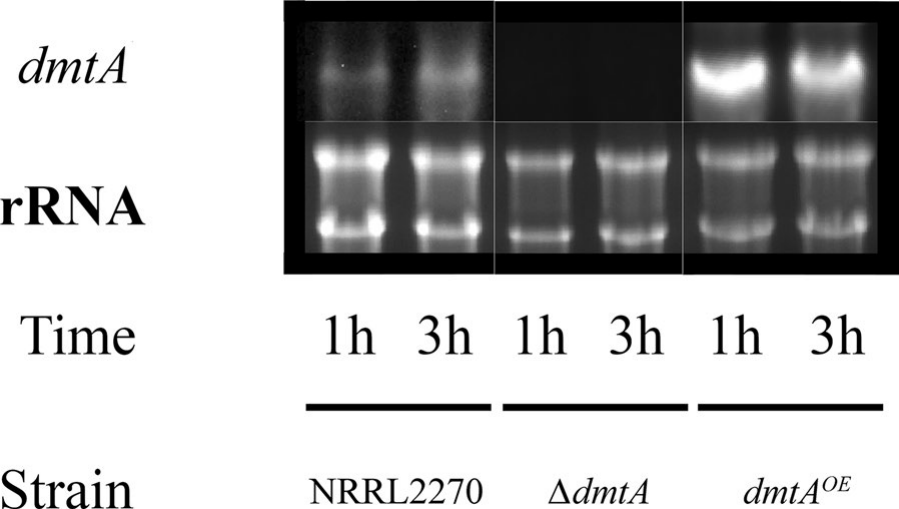
*dmtA* transcript analysis in *Aspergillus niger* strains under conditions of Mn^2+^ deficiency. RNA blot hybridisation was used to assess *dmtA* transcript levels in NRRL 2270 (parent) as well as in *dmtA*-deletion and *dmtA*-overexpressing strains. Ribosomal RNAs (5 µg per slot) provide a reference of quality and quantity of the loaded total RNA. The results shown are representative for biological duplicate analyses

Northern blot analysis revealed low expression of NRRL3_07789 in the parent strain (Fig. 1). We constructed *A. niger* strains in which NRRL3_07789 was either deleted or overexpressed under the starch-inducible glucoamylase (*glaA*) promotor [16]. In the deletion strain, no NRRL3_07789 transcript was found thus confirming the deletion of the gene. In contrast, the overexpressing strain exhibited increased NRRL3_07789 transcript level after 1 and 3 h in the manganese-limited medium.

Measurement of the Mn^2+^ intake rate of the two mutants confirmed the product of NRRL3_07789 is capable of Mn^2+^ transport. In the parent strain, an activity of 2.7 ± 0.18 pmol min^−1^ g_DCW_^−1^ was observed at Mn^2+^ concentration of 5 µg L^−1^, and it rose to > 10.0 pmol min^−1^ g_DCW_^−1^ at Mn^2+^ concentrations ≥ 100 µg L^−1^ (Table 1). The deletion mutant exhibited transport activity of < 0.2 pmol min^−1^ g_DCW_^−1^ at 5 µg Mn^2+^ L^−1^, and 0.23 and 3.6 pmol min^−1^g_DCW_^−1^ at 100 and 1000 µg L^−1^, respectively (Table 1). The strain overexpressing NRRL3_07789, however, exhibited a fivefold higher activity at 5 µg L^−1^ (13.3 pmol min^−1^g_DCW_^−1^), and this was increased to 22 and 24.9 pmol min^−1^g_DCW_^−1^ at 100 and 1000 µg Mn^2+^ L^−1^, respectively. These results indicate that NRRL3_07789 is solely responsible for the intake of Mn^2+^ ions at low concentrations, whereas a second transporter (or additional transporters) contribute to the intake of Mn^2+^ ions at high concentrations (> 100 µg L^−1^). We therefore propose that NRRL3_07789 is a divalent metal ion transporter capable of high affinity Mn^2+^transport, and name it DmtA.

**Table 1:** Specific Mn^2+^ intake in *Aspergillus niger* NRRL 2270, and the *dmtA*-deletion- and *dmtA*-overexpressing mutants

Strain	Specific Mn^2+^ ion uptake rate (pmoles min^−1^ g_DCW_^−1^)		
	5 µg L^−1^	100 µg L^−1^	1000 µg L^−1^
NRRL 2270	2.7 ± 0.2	10.0 ± 2.0	10.2 ± 2.9
*∆dmtA*	< 0.2	0.3 ± 0.1	3.6 ± 0.4
*dmtA*^*OE*^	13.3 ± 1.5	22.0 ± 3.1	24.9 ± 5.5

Specific Mn^2+^ ion intake rates in *Aspergillus niger* NRRL 2270, wild type for the *dmtA* gene, and the *dmtA*-deleted and *dmtA*-overexpressing mutants at 5, 100 and 1000 µg L^−1^ external Mn^2+^ ion concentrations. Biomass was generated on a medium with 5 µg L^−1^ Mn^2+^ (i.e., under manganese limitation) and transferred to the test media to assess decrease of the external Mn^2+^ concentrations

### Effect of *dmtA* mutations on growth of *A. niger*

**Fig. 2:**
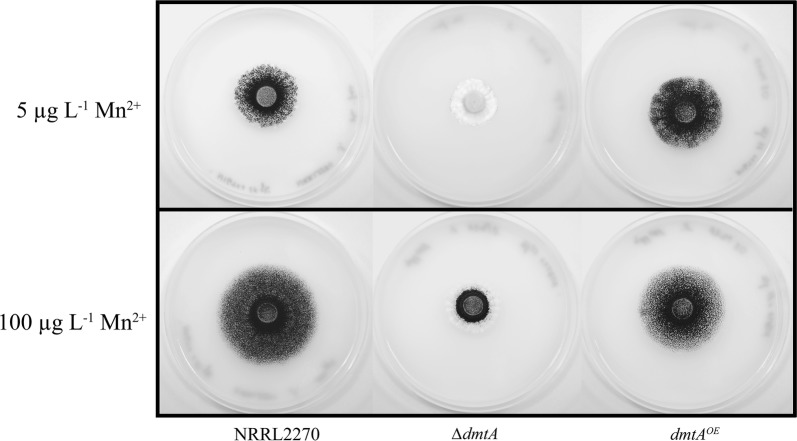
Growth phenotypes of *dmtA* mutants and wild type on minimal media with different Mn^2+^ concentrations. Radial growth was assessed on minimal medium plates with 10 g L^−1^d-glucose as the sole carbon source, pH 3.0. Plates were inoculated in triplicate (biological triplicates) and grown at 30° C. Radial growth was inspected every 24 h after the start of incubation. The Figure shows representative plates photographed against a white background after 72 h of incubation

The two mutant strains as well as the parental strain NRRL2270 were subjected to phenotypic analysis under different Mn^2+^ ion concentration. We first tested whether growth rate is influenced by the mutations of *dmtA*. Growth of the parent strain was reduced when the initial Mn^2+^ ion concentration (100 µg L^−1^) was reduced to 5 µg L^−1^ (Fig. 2), indicating that the latter concentration is correctly referred to as “suboptimal” or “limiting”. Under Mn^2+^ ion limiting conditions, the *ΔdmtA* strain started to produce mycelia only 100 h after inoculation at 5 µg L^−1^ and grew poorly at 100 µg L^−1^, indicating a major role for DmtA in providing it with this essential metal ion. The *dmtA*^*OE*^ strain at limiting Mn^2+^ ion concentrations displays growth similar to the parent at standard Mn^2+^ ion concentration (Fig. 2), implying that an enhanced activity of DmtA can efficiently import Mn^2+^ at limiting concentration of this metal ion.

### DmtA activity influences hyphal morphology

**Fig. 3:**
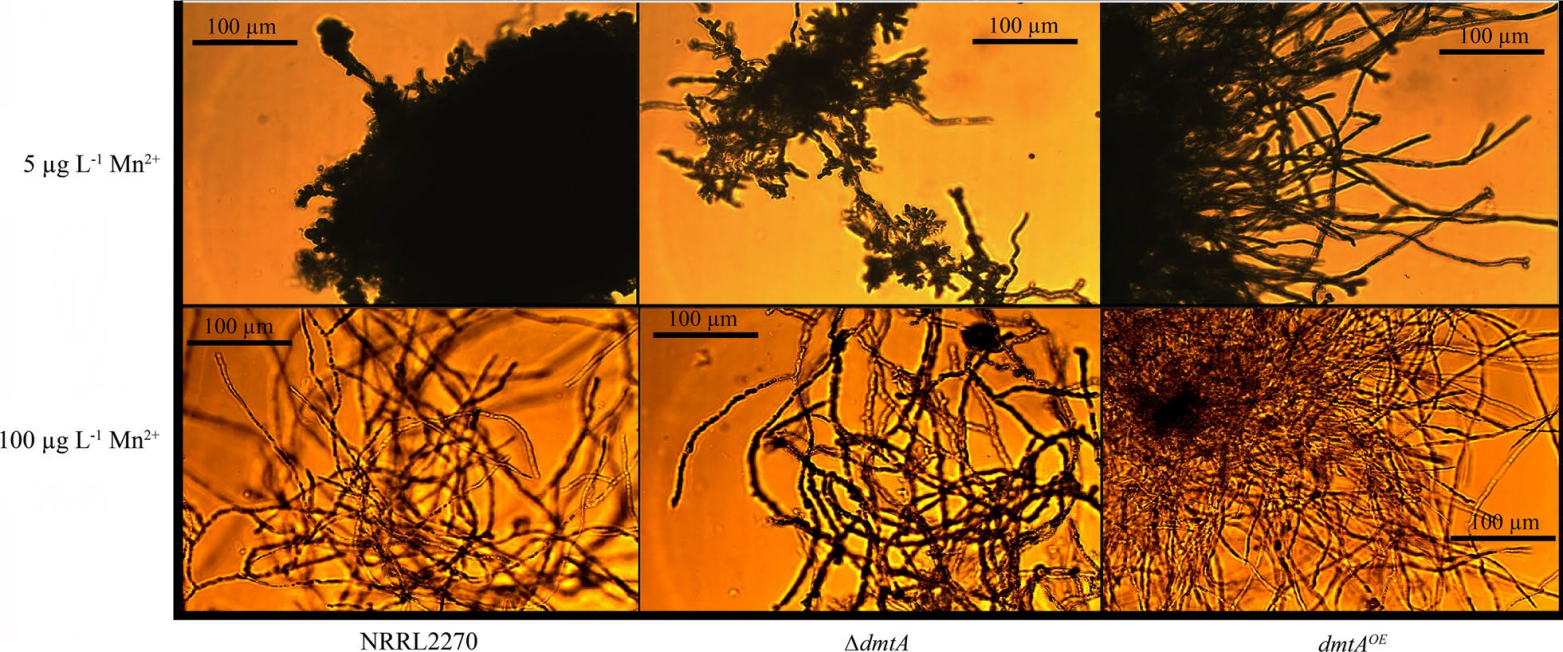
Morphology of *dmtA* mutants and wild type mycelia cultured on media with different Mn^2+^ concentrations. Citric acid production medium with two different Mn^2+^ contents (5 µg L^−1^, 100 µg L^−1^) was inoculated with conidiospores from the *dmtA* deletion strain, the *dmtA* overexpressing strain or their parent strain, NRRL 2270. Samples were taken 48 h after inoculation and mycelial pellets were visualised by microscopy

**Fig. 4:**
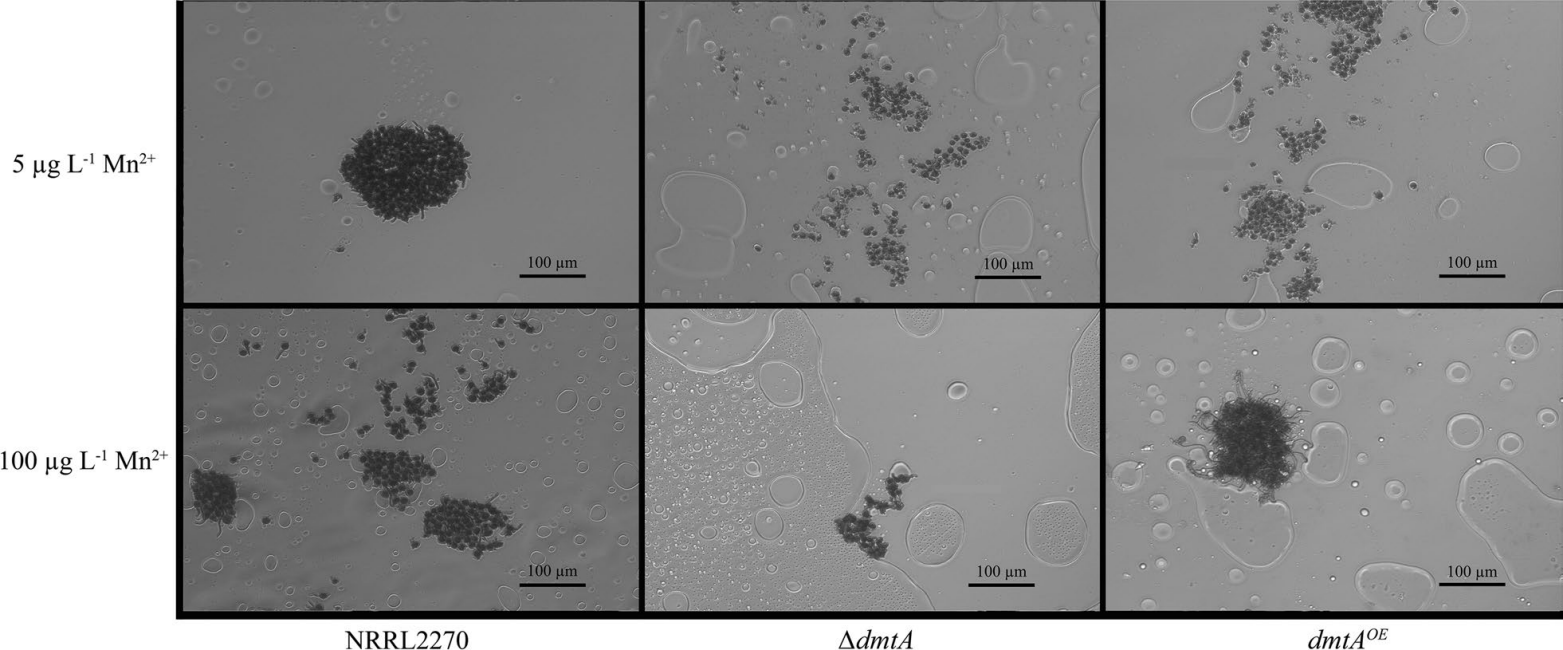
Conidiospore germination by *dmtA* mutants and wild type in media with different Mn^2+^ concentrations. Liquid cultures of *dmtA* deletion strain, *dmtA* overexpressing strain and their parent strain NRRL 2270 were initiated in citric acid production medium with two different Mn^2+^ contents (5 µg L^−1^, 100 µg L^−1^). Samples were taken 6 h after inoculation and the germinating spores were microscopically inspected, with particular attention to germtube elongation and early germling aggregation

The effect of Mn^2+^ on hyphal morphology has been documented in previous studies [17–19]. In the case of *A. niger* during citric acid hyperproduction, hyphae exhibit a swollen and highly branched form and aggregate to small and dense pellets with a smooth surface (i.e., with only core region but lacking hairy region) at limiting concentrations of Mn^2+^ (5 µg L^−1^) [20]. This phenotype was also observed in the present study with the parent strain at 5 µg L^−1^ Mn^2+^ and with the *ΔdmtA* strain under all Mn^2+^ concentrations tested (Fig. 3). The *dmtA*^*OE*^ strain did not show abnormal phenotype but exhibited long unbranched hyphae that formed fluffy pellets with large hairy region (Fig. 3). A lack of DmtA (or of Mn^2+^) also affected the rate of germination: limitation of Mn^2+^ in the medium reduced it. Deletion of *dmtA* caused the same effects even in the presence of 100 µg L^−1^ Mn^2+^, while overexpression of *dmtA* led to increased germination rate and normal hyphal morphology at limiting Mn^2+^ concentration (Table 2, Fig. 4).

**Table 2:** Germination rate of conidiospores in liquid cultures of *Aspergillus niger* strains used in this work

Strain	Germination (%)
5 µg L^−1^ Mn^2+^	
NRRL2270	33.3 ± 5.8
∆*dmtA*	30.7 ± 6.0
*dmtA*^*OE*^	42.3 ± 6.5
100 µg L^−1^ Mn^2+^	
NRRL2270	63.0 ± 5.9
∆*dmtA*	35.0 ± 6
*dmtA*^*OE*^	100 ± 0

### DmtA activity impacts citric acid overflow in A. niger

**Fig. 5:**
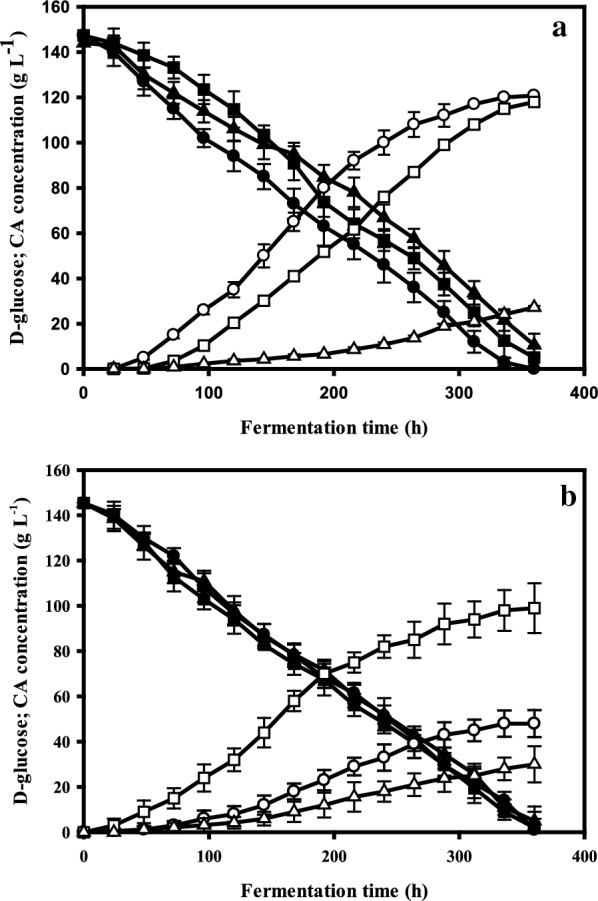
Kinetics of citrate production and d-glucose utilization on Mn^2+^-deficient- and Mn^2+^-sufficient media. Residual glucose content and citric acid production were monitored during controlled batch cultivations in fermentors. The initial concentration of d-glucose was 140 g L^−1^. **a** The fermentation kinetics under Mn^2+^ deficiency (5 µg L^−1^); **b** cultivations with sufficient Mn^2+^ (100 µg L^−1^). Fermentations were carried out in triplicate, starting from conidiospore suspensions. The investigated strains are the *dmtA* deletion mutant (∆*dmtA*), the *dmtA* overexpressing mutant (*dmtA*^*OE*^) and their parent NRRL 2270. Filled symbols represent d-glucose concentrations (●—NRRL2270, ■—*∆dmtA*, ▲—*dmtA*^*OE*^); open symbols show citric acid concentrations (○—NRRL 2270, □—*∆dmtA*, ∆—*dmtA*^*OE*^*).* Standard deviations are indicated with vertical bars for each determined concentration. Note that in the cultivations under Mn^2+^ limiting conditions (panel a), the bar is sometimes smaller than the symbol that marks the mean citric acid medium concentration

To determine the effect of a loss of *dmtA* on citric acid production in the presence of Mn^2+^ ions, we grew the parent strain, the *ΔdmtA* strain and the *dmtA*^*OE*^ strain at two different manganese concentrations, 5 and 100 µg L^−1^, in a citric-acid hyperproduction condition (medium containing 140 g L^−1^ glucose as a carbon source). Figure 5a shows that at initial concentration of 5 µg L^−1^ Mn^2+^ the parent strain produced 120 g L^−1^ citric acid after 350 h, which corresponds to a molar yield (Y_p/s_) of 0.8. The *ΔdmtA* strain produced the same amount of citric acid as the parent strain, although with a delay of about 40 h, confirming that the absence of *dmtA* has no negative effect on citric acid production level. The *ΔdmtA* strain grew slower and accumulated only about a third as much biomass as the parent strain. Consequently, its specific citric acid production (g g^−1^ biomass) is higher than in the parent strain (17.1 vs 10 g g^−1^).

**Fig. 6:**
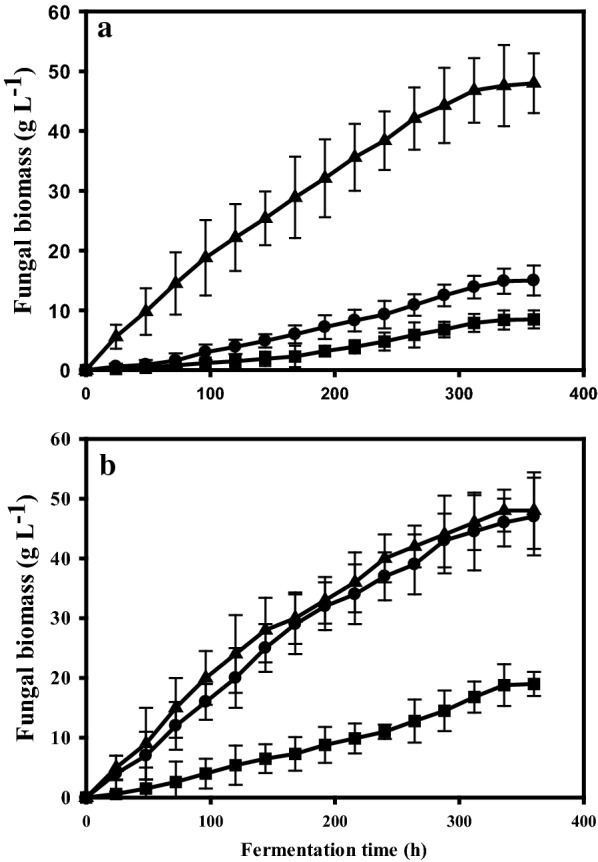
Growth kinetics of *dmtA* mutants and wild type on Mn^2+^-deficient- and Mn^2+^-sufficient media. Biomass formation was monitored during the same controlled batch cultivations as shown in Fig. 5. **a** The growth of the investigated strains on a medium with Mn^2+^ deficiency (5 µg L^−1^); **b** cultivations with sufficient Mn^2+^ (100 µg L^−1^). The investigated strains are *∆dmtA* (■), *dmtA*^*OE*^ (▲) and NRRL 2270 (●). Fermentations were carried out in triplicate, starting from conidiospore suspensions. Standard deviations are indicated with vertical bars for each determined biomass concentration (occasionally for strain *∆dmtA*, the bar is smaller than the symbol ■)

The *dmtA*^*OE*^ strain, in contrast, produced only 25–30 g L^−1^ citric acid under the same Mn-limiting conditions. This suggests that the enhanced expression of *dmtA* increases the intracellular Mn^2+^ concentration that shifts metabolism away from citric acid production. This is also reflected by the observation that the *dmtA*^*OE*^ strain forms fivefold more biomass at 5 µg L^−1^ than the parent strain (48 g L^−1^; Fig. 6a). Assuming a standard biomass yield coefficient for glucose (Y_x/s_) of 0.5, this implies that the *dmtA*^*OE*^ strain converts 68% of the provided glucose into biomass. Together with the 30 g L^−1^ citric acid, this covers for only 90% of the glucose taken up, suggesting the formation of another product (acid or polyol) in small amounts. When we looked for the presence of other metabolites known to be produced by *A. niger* (oxalic and gluconic acid, polyols) we did not find any of them in amounts > 0.1 g L^−1^ (data not shown). Hence, the carbon gap is most likely due to a lower biomass yield (Y_x/s_ < 0.5) under these conditions. While the overall d-glucose intake rate (µmoles per hour) was similar in all three cultures—resulting in similar pH profiles (data not shown)—the specific glucose intake rate (µmoles per g biomass and hour) was highest in the *ΔdmtA* and lowest in the *dmtA*^*OE*^ strain as a result of the significantly different biomass production.

Under high manganese conditions (in the presence of 100 µg L^−1^), d-glucose intake rates in the three cultures were not statistically different, whereas citric acid production was strongly influenced by mutations in *dmtA* (Fig. 5b). Citric acid production by the parent strain reached only 40–45 g L^−1^, whereas *ΔdmtA* still accumulated about 100 g L^−1^. This difference was even more dramatic when the specific production was compared (= 0.8 vs. 6.6 g g^−1^) because—although the *ΔdmtA* accumulated three-times more biomass than under Mn^2+^ limitation—the parent strain still accumulated 2.5-fold as much biomass than *ΔdmtA* (Fig. 6b). Nevertheless, these data also reveal a considerable reduction in the cells ability to produce citric acid in the presence of 100 µg L^−1^ Mn^2+^, which cannot be fully prevented by the absence of the DmtA transporter.

## Discussion

In this paper, we have identified a single NRAMP transporter gene *dmtA* in the genome of *A. niger* and provided evidence that it is of major importance for the intake of Mn^2+^ ions from the medium. Although *S. pombe* as well has a single DMT1 orthologue [10, 11], this finding was somewhat unexpected in view of the multiple genes in *S. cerevisiae* which are involved in multiple functions [21, 22]. The budding yeast Smf1p is localized in the plasma membrane, but contributes little to cellular manganese intake, whereas Smf2p is localized in the intracellular Golgi-like vesicles. However, it is the deletion of the *SMF2* gene rather than the deletion of *SMF1* that has a profound influence on cellular manganese intake [9]. The third DMT1 paralogue of budding yeast (Smf3p encoded by *SMF3*) is an iron (not manganese) transporter in the vacuolar membrane [23]. In *A. niger*, the single DmtA apparently fullfills all necessary functions required for high-affinity manganese transport. However, results from the present study do not exlude DmtA from having transport activity for other metal ions. With the data available, it is possible that *dmtA* encodes the Mn^2+^-permease characterized by Hockertz et al. [15] in *A. niger* which also transports Zn^2+^, Cu^2+^ and Cd^2+^.

Mn^2+^ transport by the *ΔdmtA* strain at low concentrations of Mn^2+^ (5 µg L^−1^) occurred at a rate that was less than 6% of that of the parent strain, whereas at 1 mg L^−1^ the rate was 30% of that of the parent strain. This confirms that *dmtA* encodes a protein capable of high-affinity Mn^2+^ transport. However, it also demonstrates that there must be at least one or more transporters for Mn^2+^ with lower affinity that contribute to a third of the intake rate at high Mn^2+^ concentrations. Indeed, a Mn^2+^ transporter with affinity in the centimolar range and which also transports Fe^2+^ (with higher affinity than Mn^2+^) has been reported by Auling [24]. As well, Netik et al. [25] showed that the citrate permease can take up Mn^2+^ complexed with citrate. In budding yeast, Mn^2+^ ions can also be imported in complex with phosphate via the Pho84 transmembrane transporter [26]. *Aspergillus niger* has a corresponding ortholog (NRRL3_00737; CBS 513.88: ANI_1_1172124; ATCC1015: ASPNIDRAFT 121846), and the operation of this mechanism would be (indirectly) supported by the finding that the detrimental effect of Mn^2+^ on citric acid accumulation can be decreased (but not eliminated) by a reduction in the concentration of inorganic phosphate in the medium. The *A. niger* Pho84 orthologue could therefore be a likely candidate for the “lower affinity” transporter detected in this study.

The effect of Mn^2+^ deficiency on citric acid accumulation and hyphal morphology has so far been considered as a consequence of insufficient availability of this metal ion. However, the data obtained with the *dmtA*^*OE*^ shed new light on this. In this mutant, cultivation at 5 µg L^−1^ Mn^2+^ ions produced the phenotypes of manganese sufficiency (low citric acid yield, filamentous morphology). This finding suggests that intracellular Mn^2+^ sufficiency—in the the *dmtA*^*OE*^ strain mediated by the increased intake rate—is more important than the concentration of Mn^2+^ in the medium in causing the effects of Mn^2+^ on citric acid accumulation and hyphal morphology. Luk and Culotta [9] showed that in *S. cerevisiae*, Smf2 functions as an intracellular Mn^2+^ transporter to deliver it to two major enzymes requiring Mn^2+^, i.e. the mitochondrially located superoxide dismutase and the Golgi-located enzymes that are involved in the glycosylation of secretory proteins. We do not know whether DmtA can fullfill this function in *A. niger*, but temporary increase in the cytosolic concentration of Mn^2+^ ions in *dmtA*^*OE*^ should lead to its enhanced availability for superoxide dismutase and the glycosylating enzymes, irrespective of the underlying mechanism.

## Conclusions

The single NRAMP divalent metal/proton symporter encoded by *dmtA* in *A. niger* is a divalent metal ion transporter capable of high-affinity manganese transport. It is of major importance for the intake of Mn^2+^ ions from the medium, and influences biomass formation rate, fungal morphology and germination of the conidiospores. Most importantly, manipulation of *dmtA* expression can modulate citric acid overflow.

## Methods

### *Aspergillus niger* strains, media and cultivation conditions

*Aspergillus niger* NRRL2270 (A60; ATCC 11414), a citric acid hyperproducer [27], was the reference strain used for this study. Strain CSFG_7001 (NRRL2270 *ΔpyrG*) was used to construct overexpression and deletion mutants (Additional file 3: Table S2). Strains were maintained on minimal medium agar plates containing 10 g d-glucose L^−1^, 6 g NaNO_3_ L^−1^, 1.5 g KH_2_PO_4_ L^−1^, 0.5 g MgSO_4_*7 H_2_O L^−1^ and 0.5 g KCl L^−1^, supplemented with 20 µL trace element solution (containing, per litre: 10 g EDTA, 4.4 g ZnSO_4_ * 7 H_2_O, 1.01 g MnCl_2_ * 4 H_2_O, 0.32 g CoCl_2_ * 6 H_2_O, 0.315 g CuSO_4_ * 5 H_2_O, 0.22 g (NH_4_)_6_Mo_7_O_24_ * 4 H_2_O, 1.47 g CaCl_2_ * 7 H_2_O, 1.1 g FeSO_4_ * 7H_2_O; [28]. The sole carbon source in this chemically defined medium optimized for citric acid production and used throughout the experiments was d-glucose at an initial level of 140 g L^−1^, and additionally contained 2.50 g (NH_4_)_2_SO_4_; 0.15 g KH_2_PO_4_; 0.15 g NaCl; 2.25 g MgSO_4_*7 H_2_O; 1.50 mg Zn^2+^; 0.10 mg Fe^2+^ and 0.06 mg Cu^2+^ per litre [29]. To control the concentration of Mn^2+^ ions in the growth medium, d-glucose was dissolved in distilled water and passed through a column (440 × 45 mm) of Dowex 50 W-X8 (100/200) cation exchange resin. All components were added to this d-glucose solution from sterile stock solutions. The final Mn^2+^ ion concentration was adjusted with MnCl_2_*4 H_2_O. All chemicals used were analytical grade and purchased from Sigma-Aldrich (Budapest, Hungary), unless specified otherwise.

Growth tests were performed on plates in medium used for submerged cultures except that initial d-glucose concentration was 10 g L^−1^. Agar is a natural gelling agent extracted from red algae enriched in essential trace elements with manganese in the mg L^−1^ range [30]. Because of this, media for growth tests were solidified with 3% agarose. For transcript analysis, replacement (transferred) cultures with d-glucose as a carbon source were used. They were performed in 500-mL Erlenmeyer flasks (VWR International Kft., Debrecen, Hungary) with 100 mL aliquots incubated at 30 °C in a rotary shaker (Infors AG, Basel, Switzerland) operating at 300 rpm. Preliminary trials had established that this rotation speed provides sufficient aeration for citric acid overflow under the given conditions. The initial pH was set at 3.0 with 3 M HCl and was not further controlled. Mycelia were pregrown for 24 h in minimal medium, and harvested by filtration on a sintered glass funnel. After a thorough wash with sterile tap water, biomass was transferred to flasks with fresh medium, containing 5 μg L^−1^ of Mn^2+^. Samples were taken 1 h and 3 h after the transfer of mycelia.

Submerged, aerobic bioreactor cultivations (henceforth referred to as fermentations) were carried out in 2.5-L glass fermentors (Sartorius AG, Göttingen, Germany) with a culture working volume of 2 L, equipped with one six-blade Rushton disc turbine impeller. Operating conditions were 30 °C and 0.75 vessel volume per minute (vvm) of aeration. The initial medium pH was adjusted to 3.0 with 3 M HCl before inoculation. The pH was measured but not controlled during fermentation. Dissolved oxygen (DO) levels were maintained at 30% saturation by appropriately adjusting the impeller tip speed. Temperature, DO, and impeller tip speed were controlled automatically by the regulatory units of the bioreactor. To minimize medium loss, the waste gas from the headspace was cooled in a reflux condenser connected to an external cooling bath (4 °C) before exiting the system. Both shake-flask cultures and fermentations were inoculated with 5 × 10^6^*A. niger* conidia per mL of medium from a freshly prepared, high-density spore suspension in a 1/10,000 Tween 20 solution.

Metal parts of the bioreactors used (stirrer attachment, aeration system, sampling tube) are built of stainless steel that may contain up to 2% of manganese [31]. Corrosion of the steel surface may lead to metal ion leaks. To monitor this, we regularly checked Mn^2+^ ion concentrations in the medium during fermentation. In addition, corrosive Mn^2+^ ion release was impeded by subjecting the bioreactor to electrochemical polishing to remove metal ions from the steel surface.

### Analytical methods

Mycelial dry cell weight (DCW) was determined from 10 mL culture aliquots as described [32]. The biomass was harvested on a pre-weighed glass wool filter and washed with tap water, after which the filter was dried at 80 °C for 1 h, until constant weight. Dry cell weight data reported in the Results are the means of two separate measurements.

Biomass yields (Y_x/s_) were calculated by dividing the amounts of the final biomass (DCW) by the total supplied carbon source (d-glucose). Specific growth rates (μ, given as the reciprocal of time, h^−1^) were calculated from the DCW increase over the time elapsed between two consecutive sampling time points; the highest of the thus obtained values was taken as the maximal specific growth rate of the culture. Likewise, d-glucose utilization rates (g L^−1^ h^−1^) were calculated from the steepest decrease in residual concentrations between two consecutive samplings.

The concentrations of d-glucose and citric acid in the growth media were determined by high-pressure/performance liquid chromatography (HPLC; Agilent Technologies 1260 Infinity II, USA) with a H^+^ exchange column (Bio-Rad Aminex HPX-87H^+^) at T = 55 °C, using isocratic elution with 10 mM H_2_SO_4_ and refractive index detection. The concentrations were calculated from two independent measurements.

To determine the cellwall-bound and intracellular manganese ion pools, fermentation broth (i.e., growth medium and mycelia) was filtered through nylon mesh, and thoroughly washed with Mn^2+^-free water to remove cellwall-bound metabolites. This washing solution was stored at − 20 °C until further use to determine cellwall-bound Mn^2+^. After removing the excess liquid by squeezing between paper sheets, mycelia were frozen in liquid nitrogen. Comminuted in liquid nitrogen and weighed, the biomass was added to Eppendorf tubes containing 700 µL sterile Mn^2+^-free water. The solution was thoroughly mixed, then spun down (11,000*g* for 10 min) to remove cellular debris. The resulting cell-free supernatant was incubated at room temperature for 30 min, and then at 100 °C for 15 min. Precipitated proteins were separated by centrifugation (20,000*g* for 10 min). The resulting clear supernatant was pipetted into Eppendorf tubes for determination of intracellular Mn^2+^. Manganese ion concentrations of both cellwall-bound and intracellular fractions were determined by inductively coupled plasma quadrupole mass spectrometry (ICP-QMS; Thermo Fisher Scientific, Bremen, Germany) equipped with Hexapole Collision Cell Technology (CCT), as described in [33]. Extracellular Mn^2+^ ion concentrations were determined from the growth medium after removal of the fungal biomass by centrifugation (10 000 *g*, 5 min).

### Manganese intake experiments

To uniformize fungal biomass for the measurements, cultures from the early growth phase were used. The inoculum was a dense suspension of mature conidiospores from spore plates with abundant Mn^2+^ in the medium. Conidiospores were inoculated in shake-flasks containing the chemically defined, citric acid producing medium with 5 µg L^−1^ Mn^2+^ (i.e., under manganese limitation) to prevent that manganese homeostasis sets in early and influence intake. When a cell concentration of ~ 1 g L^−1^ was reached—the time required for this was strain dependent—biomass was washed and transferred to the test media, where changes in the extracellular Mn^2+^ ion concentrations were monitored. The final concentrations of Mn^2+^ were adjusted to 5, 100 and 1000 μg L^−1^. Specific Mn^2+^ intake rates were calculated from the biomass-specified intake plotted against time, and were expressed in pmoles min^−1^ g_DCW_^−1^.

### Morphological studies

Fungal morphology was investigated by means of an Axio-Vision AC quantitative image analysis system. To increase contrast and visibility, lactophenol cotton blue (Fluka Chemie, Buch, Switzerland) was added to the samples to a final concentration of 10%. Stained samples were analysed under a Zeiss AxioImager phase-contrast microscope, equipped with AxioCam MRc5 camera. Samples were taken at the early exponential phase (24 h) to study cell elongation. Later samples (48 h) were taken to assess the vacuolization and swelling of the mycelia. Germination of the *A. niger* conidiospores in relation to the external manganese concentration was assessed at 6 h after inoculation, using citric acid producing medium with 10 g L^−1^d-glucose as a carbon source and Mn^2+^ concentrations of 5 and 100 μg L^−1^.

### Genomic DNA and total RNA isolation

Mycelia were harvested by filtration over nylon mesh and washed with sterile distilled water. Excess liquid was removed by squeezing between paper sheets and the biomass was quickly frozen in liquid nitrogen. For nucleic acid isolation, frozen biomass was ground to dry powder using a liquid nitrogen-chilled mortar and pestle. Genomic DNA was extracted using Promega’s Wizard SV Genomic DNA Purification System, whereas total RNA was isolated with Promega’s SV Total RNA Isolation System (Promega, Fitchburg, WI, USA).

### Northern blot analysis

Procedures applied for the quantification, denaturation, gel separation and nylon blotting of total RNA, and the subsequent hybridization of the resultant membranes with gene-specific probes (Additional file 4: Table S3) were described by Fekete et al. [34]. Five micrograms of total RNA was resolved on agarose gels. Probes were digoxigenin-labeled using the PCR DIG Probe Synthesis Kit (Roche Applied Science, Penzberg, Germany) primed with gene-specific oligonucleotide of the NRRL2270 genomic DNA. Gene-specific hybridization was visualized with a Lumi-Film Chemiluminescent Detection film (Roche Applied Science). All transcript analyses were independently repeated twice.

### Construction of deletion and overexpressing strains

We searched the *A. niger* NRRL3 genome resource at the Centre for Structural and Functional Genomics Centre using BLASTP with the *S. cerevisiae* Smf1p and Smf2p sequences (YOL122C and YHR050W, respectively) as queries. Both query sequences resulted in the identification of the same single gene, NRRL3_07789, which was termed *dmtA* (divalent metal transporter A). The CRISPR/Cas9 expression vector ANEp8_Cas9 [35] was used to clone sgRNA elements targeting the coding sequence and the promoter of the manganese transporter gene *dmtA* for gene deletion and for promoter replacement, respectively. All primers used for constructing the linear fragments and the guide sequences used for gene targeting are listed in Additional file 5: Table S4 and Additional file 6: Table S5, respectively. For overexpression, the promoter replacement cassette was constructed by fusion PCR as shown in Additional file 7: Fig. S2. Using genomic DNA of the *A*. *niger* strain NRRL2270 as template, ~ 600 bp in the upstream region and ~ 600 of the coding region of *dmtA* were amplified independently and fused by PCR to flank the glucoamylase (*glaA*) promoter using primers with complementary ends (Additional files 6 and 7: Table S5 and Fig. S2). Based on their terminal overlaps, the three fragments were joined through fusion PCR amplification, resulting in promoter replacement cassette for overexpressing *dmtA* with the *glaA* promoter. Five micrograms of the linear promoter replacement cassette was co-transformed with 500 ng of CRISPR-Cas9 plasmid targeting the promoter of *dmtA* into strain CSFG_7001 according to the transformation method described [36].

For construction of the deletion mutant, strain CSFG_7001 was transformed with 500 ng of CRISPR/Cas9 plasmid targeting the coding region of *dmtA*. Gene deletion and overexpression mutants were confirmed by PCR amplification using gene-specific primers (Additional file 5: Table S4).

### Reproducibility

Growth, intake and citric acid production data are the means of three to five independent experiments. Data were analysed and visualized with Sigmaplot software (Jandel Scientific), and for all cases standard deviations were determined. Quantitative data (n ≥ 3) were compared using ANOVA with Holm-Sidak Test for pairwise comparisons. While *p* values were often < 0.001, the criterion for significance was *p* < 0.05 in all cases.

## Supplementary information

**Additional file 1**: Figure S1. Validation of the Mn^2+^-determination assay: linearity of measured Mn^2+^ content with increasing biomass.

**Additional file 2**: Table S1. Distribution of Mn^2+^ ions in a liquid culture of *Aspergillus niger* NRRL 2270.

**Additional file 3**: Table S2: *Aspergillus niger* strains used in this study.

**Additional file 4**: Table S3: Gene-specific probes used for the transcript analysis of *Aspergillus niger dmtA* (NRRL3_07789).

**Additional file 5**: Table S4: Primers used for constructing the linear fragments.

**Additional file 6**: Table S5: CRISPR guide sequences used for targeting *dmtA* (= NRRL3_07789).

**Additional file 7**: Figure S2: Schematic illustration of construction of NRRL3_07789 promoter replacement cassette.

## Data Availability

The datasets used in the current study are available from the corresponding author on reasonable request.
