# Supplementary material for: The effects of external Mn2+ concentration on hyphal morphology and citric acid production are mediated primarily by the NRAMP-family transporter DmtA in Aspergillus niger
Source: Microb Cell Fact. 2020 Jan 30;19:17. doi: 10.1186/s12934-020-1286-7 (PMC6993379; doi:10.1186/s12934-020-1286-7)
Supplement: Supplementary file 1 — Additional file 1: Figure S1. Validation of the Mn2+-determination assay: linearity of measured Mn2+ content with increasing biomass. [file 12934_2020_1286_MOESM1_ESM.docx]

**Supplementary Figure S1. Validation of the Mn^2+^-determination assay: linearity of measured Mn^2+^ content with increasing biomass**.

Strain NRRL2770 (wild type for the NRAMP divalent metal/proton symporter gene *dmtA*) was grown in a medium with abundant Mn^2+^ (1 mg L^-1^) for 24 h in a shake flask, such that the culture was not substrate-exhausted by the time of harvest. The collected biomass was washed thoroughly with Dowex-treated water and redundant liquid was removed by pressing the mycelia between papersheets, after which it was deep-frozen in liquid nitrogen. Frozen biomass was ground to powder with liquid nitrogen-cooled mortar and pestle. Three samples of mycelial powder were taken weighing 0.1, 0.2 and 0.5 g, respectively, and cell free extracts were prepared in Eppendorf tubes using the same volume of extraction buffer for the three biomass samples. The measured Mn^2+^ concentration in the debris-cleared cell-free extracts was plotted against the mass of the mycelial samples and a straight line was fitted. The validation experiment was repeated thrice using independent cultivations (biological triplicate); the data shown are the average of the three experiments.
