# Supplementary material for: The effects of external Mn2+ concentration on hyphal morphology and citric acid production are mediated primarily by the NRAMP-family transporter DmtA in Aspergillus niger
Source: Microb Cell Fact. 2020 Jan 30;19:17. doi: 10.1186/s12934-020-1286-7 (PMC6993379; doi:10.1186/s12934-020-1286-7)
Supplement: Supplementary file 2 — Additional file 2: Table S1. Distribution of Mn2+ ions in a liquid culture of Aspergillus niger NRRL 2270. [file 12934_2020_1286_MOESM2_ESM.docx]

**Supplementary Table S1. Distribution of Mn^2+^ ions in a liquid culture of *Aspergillus niger* NRRL 2270**

| **Localization** | **Mean Mn^2+^ (%)** | **SD (%)** |
| --- | --- | --- |
| Intracellular (biomass) | 8.0 | ± 0.5 |
| Extracellular (medium) | 89.5 | ± 0.8 |
| Washing solution (cell wall matrix) | 2.5 | ± 0.2 |
| **Total culture content** | **100** | ± 1.5 |

Initial Mn^2+^ ion concentration in the culture medium was 1510 µg L^-1^ (Mn^2+^ abundancy). Three parallel shake-flask cultures (biological triplicate) were inoculated with 5×10^6^ freshly harvested conidiospores from a dense suspension in a sterile 1/10^4^ Tween 20 solution. Samples were taken after 24 h of cultivation. Each sample was measured in triplicate. Standard deviations are given at the right. For further experimental details, see Methods section.
