# Supplementary material for: The effects of external Mn2+ concentration on hyphal morphology and citric acid production are mediated primarily by the NRAMP-family transporter DmtA in Aspergillus niger
Source: Microb Cell Fact. 2020 Jan 30;19:17. doi: 10.1186/s12934-020-1286-7 (PMC6993379; doi:10.1186/s12934-020-1286-7)
Supplement: Supplementary file 3 — Additional file 3: Table S2: Aspergillus niger strains used in this study. [file 12934_2020_1286_MOESM3_ESM.doc]

**Supplementary Table S2:** *Aspergillus niger* strains used in this study

| **Strain** | **Parent** | **Genotype** | **Reference** |
| --- | --- | --- | --- |
| NRRL 2270 (ATCC 11414) | NRRL 328 (ATCC 1015) | Spontaneous derivative | Perlman et al., 1946 |
| CSFG_7001 | NRRL2270 | *ΔpyrG* | Song et al., 2018 |
| JPO_1 | CSFG_7001 | *ΔdmtA* | This study |
| JPO_2 | CSFG_7001 | *dmtA^OE^* | This study |
