# Supplementary material for: The effects of external Mn2+ concentration on hyphal morphology and citric acid production are mediated primarily by the NRAMP-family transporter DmtA in Aspergillus niger
Source: Microb Cell Fact. 2020 Jan 30;19:17. doi: 10.1186/s12934-020-1286-7 (PMC6993379; doi:10.1186/s12934-020-1286-7)
Supplement: Supplementary file 4 — Additional file 4: Table S3: Gene-specific probes used for the transcript analysis of Aspergillus niger dmtA (NRRL3_07789). [file 12934_2020_1286_MOESM4_ESM.doc]

**Supplementary Table S3:** Gene-specific probes used for the transcript analysis of *Aspergillus niger* *dmtA* (NRRL3_07789).

| **Primer** |  | **Sequence (5’-3’)** |
| --- | --- | --- |
| *A. niger*  NRRL3_07789 | Forward | TTTCGCTATGCTTTGCTCTTC |
|  | Reverse | TACAGGGAAGCACCAGCAAC |
