# Supplementary material for: The effects of external Mn2+ concentration on hyphal morphology and citric acid production are mediated primarily by the NRAMP-family transporter DmtA in Aspergillus niger
Source: Microb Cell Fact. 2020 Jan 30;19:17. doi: 10.1186/s12934-020-1286-7 (PMC6993379; doi:10.1186/s12934-020-1286-7)
Supplement: Supplementary file 5 — Additional file 5: Table S4: Primers used for constructing the linear fragments. [file 12934_2020_1286_MOESM5_ESM.doc]

**Supplementary Table S4:** Primers used for constructing the linear fragments.

| **Primer name** | **Sequence (5’ to 3’ oriented)** | **Used for:** |
| --- | --- | --- |
| Fw_LIC2 | CAACCTCCAATCCAATTTGACTCCGCCGAACGTACTG | Amplification of CRISPR gRNAs to clone into the Cas9 plasmid |
| Rev_LIC2 | ACTACTCTACCACTATTTGAAAAGCAAAAAAGGAAGGTACAAAAAAGC | Amplification of CRISPR gRNAs to clone into the Cas9 plasmid |
| Rev_gRNACDS_07789 | TCGCTAACCATTTGCCCAGCGACGAGCTTACTCGTTTCG | Construction of CRISPR gRNA targeting coding region of *dmtA* |
| Fw_gRNACDS_07789 | GCTGGGCAAATGGTTAGCGAGTTTTAGAGCTAGAAATAGCAAG | Construction of CRISPR gRNA targeting coding region of *dmtA* |
| Fw_gRNApromo_07789 | TTCCCTCTCCTGCTTCCGACGTTTTAGAGCTAGAAATAGCAAG | Construction of CRISPR gRNA targeting promoter of *dmtA* |
| Rev_gRNApromo_07789 | TCGGAAGCAGGAGAGGGAAGACGAGCTTACTCGTTTCG | Construction of CRISPR gRNA targeting promoter of *dmtA* |
| Fw_5’promo_07789 | GCTTATCGAATCAGAGGGAAG | Amplification of upstream region of promoter *dmtA* for *dmtA* promoter replacement cassette construction |
| Rv_5’promo_07789 | GGCGGACTGAGAAGCTC | Amplification of upstream region of promoter *dmtA* for *dmtA* promoter replacement cassette construction |
| Fw_CDS_07789 | ATGAATTGTCCTTCGCG | Amplification of coding region of *dmtA* for *dmtA* promoter replacement cassette construction |
| Rv_CDS_07789 | GGAGTAGTTTCCCGGATC | Amplification of coding region of *dmtA* for *dmtA* promoter replacement cassette construction |
| Fw_glaA_07789 | CTGAAGAGCTTCTCAGTCCGCCGA GCTAGCCGAGAGCAG | Amplification of *glaA* promoter for *dmtA* promoter replacement cassette construction |
| Rv_glaA_07789 | CGGTGCGCGAAGGACAATTCATTGCTGAGGTGTAATGATGC | Amplification of *glaA* promoter for *dmtA* promoter replacement cassette construction |
| Fw_screeningKO | AGAATCTCGCGGTTCCAGAC | Confirmation of deletion mutant of *dmtA* |
| Rv_screeningKO | GAGCTCGATGGCTGATAGTG | Confirmation of deletion mutant of *dmtA* |
| Fw_screeningOE | CGGTGACTGCCCATCTAG | Confirmation of overexpressing mutant of *dmtA* |
| Rv_screeningOE | GAAACCAGCTTAGCACCAG | Confirmation of overexpressing mutant of *dmtA* |
|  |  |  |
