# Supplementary material for: The effects of external Mn2+ concentration on hyphal morphology and citric acid production are mediated primarily by the NRAMP-family transporter DmtA in Aspergillus niger
Source: Microb Cell Fact. 2020 Jan 30;19:17. doi: 10.1186/s12934-020-1286-7 (PMC6993379; doi:10.1186/s12934-020-1286-7)
Supplement: Supplementary file 6 — Additional file 6: Table S5: CRISPR guide sequences used for targeting dmtA (= NRRL3_07789). [file 12934_2020_1286_MOESM6_ESM.doc]

**Supplementary Table S5:** CRISPR guide sequences used for targeting *dmtA* (= NRRL3_07789).

| **Guide ID** | **Targeted region** | **Genomic target sequence (5'> 3')** | **PAM** |
| --- | --- | --- | --- |
| NRRL3_07789_CDS_G1 | Coding region of NRRL3_07789 | GCTGGGCAAATGGTTAGCGA | GGG |
| NRRL3_07789_Promo-G1 | Promoter of NRRL3_07789 | TTCCCTCTCCTGCTTCCGAC | TGG |
