# Supplementary material for: The effects of external Mn2+ concentration on hyphal morphology and citric acid production are mediated primarily by the NRAMP-family transporter DmtA in Aspergillus niger
Source: Microb Cell Fact. 2020 Jan 30;19:17. doi: 10.1186/s12934-020-1286-7 (PMC6993379; doi:10.1186/s12934-020-1286-7)
Supplement: Supplementary file 7 — Additional file 7: Figure S2: Schematic illustration of construction of NRRL3_07789 promoter replacement cassette. [file 12934_2020_1286_MOESM7_ESM.pptx]

## Slide 1
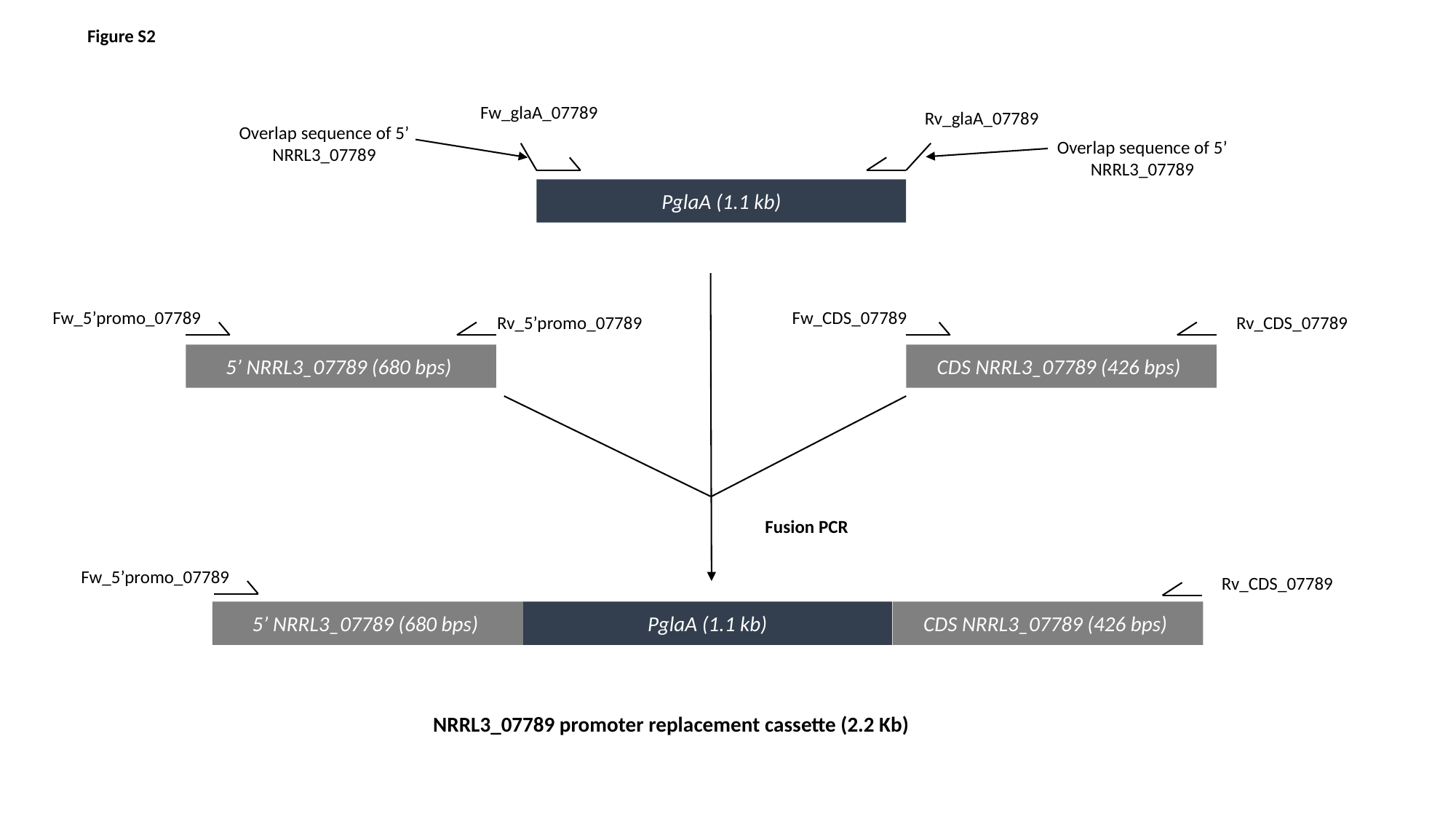

Figure S2
Fw_glaA_07789
Rv_glaA_07789
Overlap sequence of 5’ NRRL3_07789
Overlap sequence of 5’ NRRL3_07789
PglaA (1.1 kb)
Fw_CDS_07789
Rv_5’promo_07789
Rv_CDS_07789
5’ NRRL3_07789 (680 bps)
CDS NRRL3_07789 (426 bps)
Fusion PCR
Fw_5’promo_07789
Rv_CDS_07789
5’ NRRL3_07789 (680 bps)
PglaA (1.1 kb)
CDS NRRL3_07789 (426 bps)
NRRL3_07789 promoter replacement cassette (2.2 Kb)
Fw_5’promo_07789
